# Associations between Sleep-Disordered Breathing and Metabolic Risk Factors beyond Obesity

**DOI:** 10.1155/2018/1567683

**Published:** 2018-10-22

**Authors:** Yusuke Wakabayashi, Rie Oka, Masako Nakaya, Shigehiro Karashima, Mitsuhiro Kometani, Masaru Sakurai, Kenichi Yoshimura, Takashi Yoneda

**Affiliations:** ^1^Department of Internal Medicine, Hokuriku Central Hospital, Toyama, Japan; ^2^Internal Medicine, Kanazawa University Graduate School of Medical Science, Kanazawa, Japan; ^3^Department of Epidemiology and Public Health, Kanazawa Medical University, Japan; ^4^Department of Biostatistics, Innovative Clinical Research Center (iCREK), Kanazawa University Hospital, Kanazawa, Japan

## Abstract

**Objective:**

Individuals with multiple metabolic risk factors often experience concomitant sleep-disordered breathing (SDB). We aimed to determine the associations of SDB with individual components of metabolic syndrome independent of obesity.

**Methods:**

A cross-sectional study was conducted in 1137 employees aged 30–64 years. Apnea-hypopnea index (AHI) was assessed using a portable monitor for obstructive sleep apnea by admission. Of these, 451 participants took an oral glucose tolerance test to assess homeostatic model assessment of insulin resistance (HOMA-IR) and Matsuda insulin sensitivity index (ISI).

**Results:**

The odds ratio (OR) of the highest category of the AHI (≥15 episodes per hour) compared to the lowest one (<5 episodes per hour) was significantly elevated for hypertension, for hypertriglyceridemia, and for low HDL-cholesterolemia when adjusted for age, sex, and alcohol and smoking status (*p* < 0.05). After further adjustment for body mass index (BMI) or waist circumference, the associations for hypertension still remained statistically significant (*p* < 0.05) while those for hypertriglyceridemia and low HDL-cholesterolemia were no longer significant. The association between higher insulin resistance as assessed by HOMA-IR and Matsuda ISI and higher categories of the AHI was also lost after adjustment for BMI.

**Conclusion:**

Obesity was a strong confounding factor in the association between SDB and most metabolic risk factors including insulin resistance, except for hypertension. Further longitudinal study is needed to examine the temporal or causal relationships between SDB and metabolic risk factors. This trial is registered with UMIN-CTR UMIN000028067.

## 1. Introduction

Obesity, particularly abdominal obesity, can cause individuals to develop multiple metabolic disorders including dyslipidemia, hyperglycemia, and/or hypertension. Clustering of metabolic risk factors is not incidental, but rather evidence of “metabolic syndrome” or “syndrome X” [1]. Individuals with “syndrome X” often experience concomitant sleep-disordered breathing (SDB) which acts synergistically to increase their risk for cardiovascular disease [2, 3]. Thus, some investigators have suggested that “syndrome X” may include SDB and must then be called “syndrome Z” [4].

Recently, SDB has been reported to be independently associated with metabolic syndrome [5, 6] and with its more fundamental factor, insulin resistance [7–9]. However, because both SDB and metabolic derangements are strongly correlated with indices of obesity, obesity becomes an important confounder in the relationship between SDB and metabolic abnormalities. It is unknown whether SDB is causally related to metabolic abnormalities or is just a bystander in the relationships between obesity and metabolic abnormalities.

The aim of this study was to determine the associations of SDB with individual components of metabolic syndrome independent of obesity. The degrees of insulin resistance were also examined in relation to the severity of SDB.

## 2. Materials and Methods

### 2.1. Participants

Participants included Japanese public school employees who received medical checkups at the Hokuriku Central Hospital between April 2006 and March 2010. On checkups, we recommended to take a sleep study to employees with sleep-related symptoms of obstructive sleep apnea including loud snoring, witnessed pauses in breathing, restless sleep, morning headaches, and/or daytime sleepiness or fatigue, unless they had already been treated for sleep apnea syndrome. Also, excluded from the study were those individuals who had <4 hours of quality data on record or missing data. Additionally, some participants spent one more night (total 2 nights) at the hospital and underwent an oral glucose tolerance test (OGTT) after an overnight fast, as previously reported [10]. Participants were considered smokers if they smoked at least 1 cigarette per day. Alcohol use was defined by the number of days per week it was consumed, regardless of the amount. Informed consent was obtained via an opt-out method, and the Institutional Review Board of the Kanazawa University approved the study protocol on June 21, 2017 (IRB no. 2497-1); the study protocol conformed to the provisions of the Declaration of Helsinki. The study was registered on the University Hospital Medical Information Network Clinical Trials Registry (UMIN-CTR, http://www.umin.ac.jp/ctr, UMIN ID: UMIN000028067).

### 2.2. Blood Sampling and Anthropometric Measurements

After an overnight fast, blood samples were drawn from the antecubital vein to measure triglycerides, high-density lipoprotein (HDL) cholesterol, and fasting plasma glucose. Triglycerides and HDL cholesterol were measured using enzymatic analytical chemistry (Autoanalyzer BioMajesty JCA-BM1650, JEOL Ltd., Tokyo, Japan), and plasma glucose was assessed using the glucose oxidase method (Automatic Glucose Analyzer ADAMS Glucose GA-1160, Arkray, Kyoto, Japan) in the hospital laboratory. Insulin concentration assays were performed by the chemiluminescence immunoassay method at a commercial laboratory (BML Inc., Tokyo, Japan), with an intra-assay coefficient of variation (CV) of 2.4–3.2% based on 10 replicates of 3 different samples. Resting blood pressure was measured in the sitting position with an automatic device (Colin Model BP-203RV, Colin, Tokyo, Japan) after ≥5 minutes of rest. Measurements of body mass index (BMI) and waist circumference were conducted according to published methods [11].

### 2.3. Assessment of Metabolic Risk Factors and Calculation of Indices of Insulin Resistance

Participants were assessed with metabolic risk factors according to the following definitions: hypertension, a systolic/diastolic blood pressure ≥ 140/90 mmHg and/or taking antihypertensive medication; hypertriglyceridemia, triglycerides ≥ 150 mg/dL (1.69 mmol/L); low HDL-cholesterolemia, <40 mg/dL (1.04 mmol/L) for men and <50 mg/dL (1.30 mmol/L) for women; and impaired fasting plasma glucose (IFG), ≥110 mg/dL (6.1 mmol/L) [12]. Obesity was defined by BMI ≥ 25.0 kg/m^2^ according to the Asian criterion of obesity [13]. The indices of insulin resistance were as follows: Matsuda insulin sensitivity index (ISI) = 10000/(Glu_0_ × Ins_0_ × Glu_120_ × Ins_120_)^0.5^ and homeostatic model assessment of insulin resistance (HOMA-IR) = Glu_0_ × Ins_0_/405 [14], where Glu_*x*_ and Ins_*y*_ represented values at time *x* or *y* (min) during the OGTT.

### 2.4. Sleep Study

Sleep studies were conducted at the Health Check Department of Hokuriku Central Hospital for 1 night admission. Each participant's apnea-hypopnea index (AHI) was assessed using the PulSleep LS-100 (Fukuda Denshi, Tokyo, Japan) which digitally records nasal airflow via nasal cannula, oxygen saturation, and snoring sounds. AHI was defined as the average number of apneic plus hypopneic episodes per hour of sleep. Apnea was defined as a complete cessation of nasal airflow, and hypopnea was defined as a decrease in nasal airflow of at least 50% of baseline for ≥10 seconds. Both apnea and hypopnea must be by ≥4% decrease in oxygen saturation [15].

### 2.5. Statistical Analysis

All analyses were conducted using SPSS software version 24.0 for Windows (SPSS Inc., Chicago, IL, USA). Participants were classified using the AHI at commonly used clinical cut-off points. Three severity gradients or categories were used for the AHI in this study: none/minimal (<5 episodes per hour (reference)), mild (≥5 but <15 per hour), and moderate-to-severe (≥15 per hour). Data are presented as mean ± SD values for continuous variables and as a proportion for categorical variables. Tests to identify a linear trend across categories were performed by assigning the median value within each category and treating the categories as a continuous variable. Binary logistic regression analyses were performed to estimate the adjusted odds ratio (OR) for having metabolic risk factors in each category relative to the reference category (AHI < 5.0). The following covariates were used: age, sex, smoking (a 3-level variable: current, former, and never smoker), and alcohol use (a 3-level variable: drinking every day, drinking 1–6 days per week, and drinking <1 day per week). First, these nonadipose covariates were first included in the regression model. Subsequently, to assess whether the association between the stage of sleep disorder and each metabolic risk factor was independent of obesity, either BMI or waist circumference was additionally included in the model. Because the distribution of waist circumference was different between men and women, waist circumference was standardized to a mean of 0 and standard deviation of 1 in men and women, respectively, before the inclusion in the multiple regression model. Finally, in 451 subjects undertaking the OGTT, the levels of HOMA-IR and Matsuda ISI were compared among three different categories of the AHI. The comparisons were performed before and after adjustments for BMI, using analysis of covariance (ANCOVA). Analyses of HOMA-IR were conducted after logarithmic transformation because of their skewed distribution. A *p* value of <0.05 was considered statistically significant.

## 3. Results

The overall study participants were composed of 1173 subjects with a mean age of 51.1 ± 7.2 years and a mean BMI of 24.7 ± 3.5 kg/m^2^. Characteristics of the study participants according to AHI categories are presented in Table 1. Compared with those with lower AHI values, participants with higher AHI values showed a significantly higher proportion of men and significantly higher levels of BMI, blood pressure, fasting plasma glucose, triglycerides, and HOMA-IR and lower levels of HDL cholesterol and Matsuda ISI (*p* for the trend < 0.01). The proportions of smokers, daily drinkers, and those taking antihypertensive drugs were significantly higher in those with higher AHI values (*p* < 0.05).

**Figure 1:**
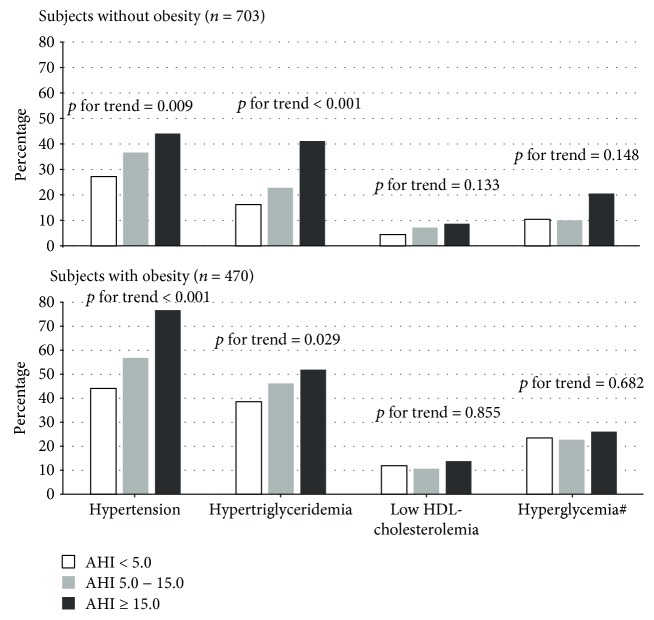
Prevalence of metabolic abnormalities by an AHI category stratified by obesity, using BMI ≥ 25.0 kg/m^2^ as a cut-off. ^#^Hyperglycemia was defined as fasting plasma glucose ≥ 110 mg/dL (6.1 mmol/L) and/or taking medications for diabetes. AHI: apnea-hypopnea index; BMI: body mass index; HDL: high-density lipoprotein; NS: not statistically significant.

Figure 1 shows the prevalence of metabolic abnormalities by an AHI category stratified by obesity. For both strata, the proportions of participants with hypertension and hypertriglyceridemia were significantly higher in higher categories of the AHI showed (*p* for the trend < 0.05). In participants without obesity, the prevalence of low HDL-cholesterolemia and hyperglycemia tended to be higher in higher categories of the AHI but the trend test did not reach statistical significance.

Table 2 shows the ORs for having individual risk factors according to the level of sleep-disordered breathing. When adjusted for age, sex, and alcohol and smoking status, the ORs of the highest category of the AHI were significantly elevated for hypertension, for hypertriglyceridemia, and for low HDL-cholesterolemia (*p* < 0.05). After further adjustment for BMI, the associations for hypertension still remained statistically significant (*p* < 0.05) while those for hypertriglyceridemia and low HDL-cholesterolemia were no longer significant. The elevated ORs for hypertension in the highest category of the AHI were also significant after adjustments for waist circumference in place of BMI (*p* < 0.05).

**Figure 2:**
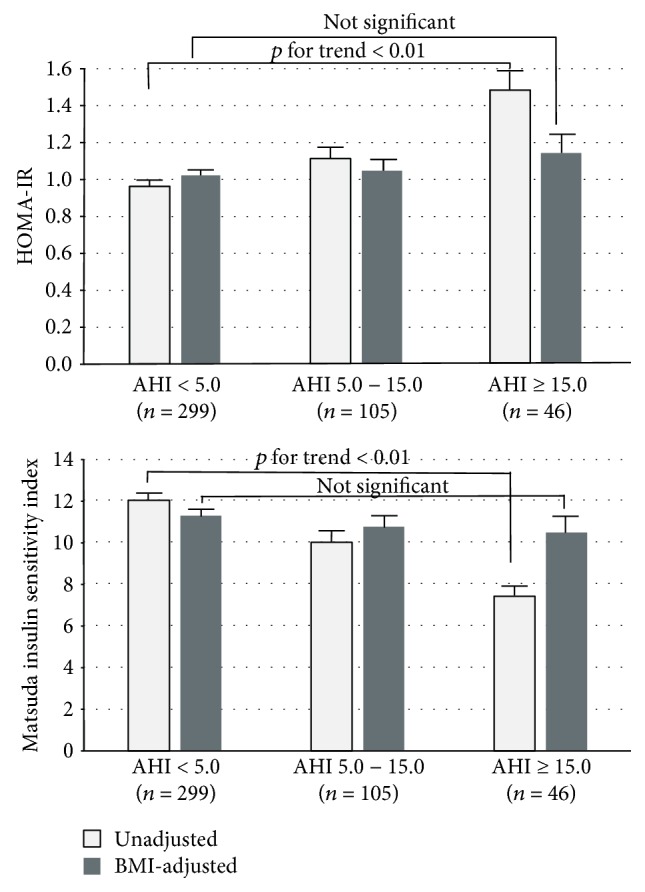
Unadjusted and BMI-adjusted indices of insulin resistance by an AHI category. HOMA-IR was log-transformed prior to analysis to reduce skewedness, and calculated values were untransformed after analysis. Error bars show standard error of the mean. AHI: apnea-hypopnea index; BMI: body mass index; HOMA-IR: homeostatic model assessment of insulin resistance.

Finally, the levels of indices of insulin resistance were compared among categories of the AHI in 451 subjects (Figure 2). HOMA-IR was significantly higher, and Matsuda ISI was significantly lower in higher categories of the AHI (*p* for the trend < 0.01), but after adjusted for BMI, both indices no longer showed a significant trend, indicating that the association between AHI severity and insulin resistance was confounded by BMI. Adjustments for waist circumference in place of BMI showed similar results (data not shown). These 451 subjects took the OGTT not for any clinical signs but for financial reasons; they were slightly older (52.5 ± 6.7 yrs vs. 50.2 ± 7.4, *p* < 0.05), and the proportion of men was higher (80.0% vs. 73.1%, *p* < 0.05) than those who did not take the OGTT, but other characteristics including BMI was not significantly different.

## 4. Discussion

In this study, the independent association between severity of SDB and each metabolic risk factor was cross-sectionally examined in middle-aged Japanese men and women. After controlling for obesity, independent association was found for hypertension but not for dyslipidemia, hyperglycemia, and insulin resistance. These results suggest that SDB is an independent determinant for hypertension but may be only a bystander in the link between obesity and other metabolic abnormalities.

The link between SDB and hypertension was independent of obesity in this study, consistent with large community-based studies [6, 16, 17] and several randomized trials [18]. Their link was maintained after adjustment for waist circumference, an index of abdominal obesity, as well as BMI, an index of generalized obesity. In the CIRCS study, which demonstrated an independent association between SDB and metabolic syndrome in nonoverweight Japanese, the OR for hypertension in subjects with ≥15 hypoxia events/hour was highest (OR, 2.5; 95% CI, 1.4–4.6) among ORs for all components of metabolic syndrome [19]. Their results are in line with our observation that the OR for hypertension was only significantly elevated after controlling for obesity but not those for other metabolic risk factors. Although the effects of SDB on blood pressure have been reported to be weakened in elder subjects, at least in relatively younger subjects including our population, SDB seems to be an independent contributor to elevated blood pressure beyond obesity.

The association between hypertriglyceridemia and higher categories of the AHI was disappeared after adjustments for BMI in this study. There have been two large studies demonstrating an association between SDB and metabolic syndrome conducted in Japan [5, 6]. However, looked into the individual components of metabolic syndrome in these studies, after controlling for concomitant obesity, the association between hypertriglyceridemia and severity of SDB was diminished in one study [5] and was maintained only in nonoverweight subjects in the other with OR of 1.7 and 95% CI of 1.0–2.8 [6]. Moreover, the effects of OSA-targeted therapeutic intervention using CPAP on the lipid profile are controversial among investigators [20]. A metaregression analysis including 29 studies with 1958 subjects has concluded that CPAP treatment for OSA decreased total cholesterol and LDL cholesterol and did not affect TG levels [21]. Because hypertriglyceridemia is the strongest correlate with abdominal obesity among metabolic risk factors [22], the magnitude of association independent of obesity, if any, appears to be small relative to other components such as hypertension.

The prevalence of hyperglycemia, as assessed by fasting plasma glucose ≥110 mg/dL, was not significantly increased in higher categories of the AHI after controlling for BMI, consistent with prior studies [5, 6, 23]. The association between IFG and SDB was insignificant after adjustments for anthropometric indices in 1344 subjects from the Korean genome and epidemiology study [23] and in a male working population [5]. Although some prospective studies have demonstrated that SDB independently preceded and predicted the development of type 2 diabetes [19, 24, 25], even in these cases, the cross-sectional associations at baseline between SDB and diabetes after controlling for BMI [19, 25] and between SDB and fasting glucose levels [24] were not significant.

Why was the independent association between metabolic risk factors and SDB not seen in our study? In the CIRCS Study, the association between glucose abnormality and SDB after controlling for BMI was significant only in overweight subjects with OR of 1.6 and 95% CI of 1.0–2.6 and not in nonoverweight individuals with OR of 1.4 and 95% CI of 0.8–2.5 [6]. In study populations composed of less overweight subjects like this study, it may be difficult to detect the independent association between glucose abnormality and SDB. Further study is needed, comprising subjects without obesity and with SDB.

Furthermore, we failed to show the higher levels of insulin resistance in relation to severity of AHI categories after controlling for BMI. The discordance with prior epidemiological and clinical studies [7–9] may be attributed to relatively younger age of our study subjects. Because obesity is a major determinant of insulin resistance in younger subjects, a marginal influence of SDB in insulin resistance would be difficult to be demonstrated. Confounding effects of obesity also render it difficult to demonstrate an independent effect of CPAP therapy on insulin sensitivity. In a study of 40 treatment-naïve, nondiabetic German subjects, insulin sensitivity as assessed by a hyperinsulinemic euglycemic clamp was not significantly improved in patients with obesity [26]. Negative effects of CPAP therapy on insulin resistance have been also reported in two previous randomized controlled trials in patients with obesity [27, 28] and in a recent meta-analysis [29].

The strength of this study was the simultaneous evaluation of multiple metabolic risk factors in relation to the severity of SDB, but several limitations should be considered. First, the measurement of blood pressure was performed approximately at 9 o'clock in the daytime. Sasaki et al. reported that approximately half the OSAS patients displayed morning hypertension [30], which may be missed by our evaluation. Second, insulin resistance was not measured by the glucose clamp technique, which is the gold standard for evaluating insulin resistance/sensitivity; however, it has been demonstrated that Matsuda ISI and HOMA-IR correlated well with directly measured insulin resistance and with metabolic abnormalities in nondiabetic subjects [31]. Third, obesity was assessed only by BMI and waist circumference. However, these two anthropometric indices are not inferior to visceral adipose tissue in correlation with insulin resistance [32] or with blood pressure [22] in Japanese men and women. Fourth, the sleep device in this study did not measure chest or abdomen movements, by which the strict differentiation between obstructive and central type of SDB was difficult. Finally, the cross-sectional design does not allow examination of the temporal or causal relationships between SDB and metabolic risk factors. Longitudinal studies are needed to confirm whether SDB is one of the secondary causes of incident hypertension in this population.

In conclusion, the cross-sectional associations of SDB with metabolic abnormalities vary across the individual risk factors. Obesity was a strong confounding factor in the association between SDB and most metabolic risk factors including insulin resistance. However, for hypertension, SDB had an independent association beyond confounding effects of obesity. Further longitudinal study is needed to examine the temporal or causal relationships between SDB and metabolic risk factors.

## Figures and Tables

**Table 1:** Basic clinical characteristics of the study subjects according to the level of the apnea-hypopnea index (AHI).

	Overall (*N* = 1173)	AHI			*p* for the trend
		<5.0 (*N* = 812)	5.0–15.0 (*N* = 250)	≥15.0 (*N* = 111)	
Age (years)	51.1 ± 7.2	50.5 ± 7.3	52.9 ± 6.1	51.2 ± 8.0	0.02
Male gender (%)	75.8	69.5	86.4	98.2	<0.01
Anthropometries					
Body mass index (BMI) (kg/m^2^)	24.7 ± 3.5	23.9 ± 3.0	25.8 ± 3.4	28.1 ± 4.6	<0.01
Obesity (%)^∗^	40.1	31.0	56.4	69.4	<0.01
Waist circumference (cm) in men	86.5 ± 8.8	84.2 ± 7.5	89.0 ± 8.2	93.5 ± 10.6	<0.01
Waist circumference (cm) in women	81.5 ± 9.4	80.8 ± 8.9	86.1 ± 11.6	93.5 ± 13.4	<0.01
Metabolic parameters					
Systolic blood pressure (mmHg)	131.5 ± 19.0	128.4 ± 18.0	135.7 ± 17.9	144.6 ± 21.4	<0.01
Diastolic blood pressure (mmHg)	82.0 ± 11.8	80.1 ± 11.3	84.7 ± 11.2	89.8 ± 11.8	<0.01
Fasting plasma glucose (mg/dL)	100.8 ± 17.9	99.8 ± 18.4	102.8 ± 17.4	103.6 ± 15.2	0.01
Triglycerides (mg/dL)	78/109/156	73/102/145	85/125/184	104/146/193	<0.01
HDL cholesterol (mg/dL)	58.0 ± 14.2	60.0 ± 14.8	54.4 ± 12.4	51.4 ± 9.9	<0.01
HOMA-IR (*N* = 451)	0.8/1.0/1.5	0.7/1.0/1.4	0.8/1.1/1.7	1.0/1.4/2.1	<0.01
Matsuda ISI (*N* = 451)	11.0 ± 8.7	12.0 ± 8.9	10.0 ± 9.0	7.3 ± 4.9	<0.01
Habits					
Smokers (%)	19.6	18.1	22.0	25.2	0.04
Daily drinkers (%)	33.7	31.9	36.0	41.4	0.03
Taking medications					
Antihypertensive drugs (%)	15.0	12.6	17.2	27.9	<0.01
Lipid-lowering drugs (%)	7.5	6.5	10.4	8.1	0.24
Glucose-lowering drugs (%)	4.1	3.6	5.2	5.4	0.24

Data are expressed as mean ± SD, 25/50/75th percentile value, or number (%). ^∗^Obesity was defined by BMI ≥ 25.0 kg/m^2^. HDL cholesterol: high-density lipoprotein cholesterol; HOMA-IR: homeostatic model assessment of insulin resistance; Matsuda ISI: Matsuda insulin sensitivity index.

**Table 2:** Multivariable analysis for metabolic abnormalities according to the apnea-hypopnea index (AHI) category.

	Odds ratios (95% CI) by an AHI category		
	<5.0 (*N* = 812)	5.0–15.0 (*N* = 250)	≥15.0 (*N* = 111)
Hypertension			
Crude	1.0	**1.9 (1.4–2.6)** ^∗^	**4.2 (2.7–6.3)**
Multivariable-adjusted^†^	1.0	**1.6 (1.2–2.1)**	**3.6 (2.3–5.6)**
Multivariable and BMI-adjusted	1.0	1.1 (0.8–1.6)	**1.9 (1.2–3.1)**
Multivariable and waist-adjusted	1.0	1.1 (0.8–1.6)	**2.1 (1.3–3.3)**
Hypertriglyceridemia^‡^			
Crude	1.0	**1.9 (1.4–2.5)**	**3.1 (2.1–4.7)**
Multivariable-adjusted	1.0	**1.6 (1.1–2.2)**	**2.4 (1.6–3.7)**
Multivariable and BMI-adjusted	1.0	1.1 (0.8–1.6)	1.3 (0.8–2.0)
Low HDL-cholesterolemia			
Crude	1.0	1.4 (0.8–2.3)	1.8 (1.0–3.5)
Multivariable-adjusted	1.0	1.5 (0.9–2.6)	**2.2 (1.1–4.4)**
Multivariable and BMI-adjusted	1.0	1.2 (0.7–2.1)	1.4 (0.6–2.9)
Hyperglycemia^‡^			
Crude	1.0	1.2 (0.8–1.8)	**1.9 (1.2–3.0)**
Multivariable-adjusted	1.0	1.0 (0.7–1.5)	1.6 (1.0–2.6)
Multivariable and BMI-adjusted	1.0	0.7 (0.4–1.0)	0.7 (0.4–1.2)

^∗^Bold font indicates that the odds ratio is statistically significant (*p* < 0.05). ^†^Multivariable model adjusted for age, sex, alcohol intake, and smoking status. ^‡^Hyperglycemia was defined as fasting plasma glucose ≥ 110 mg/dL (6.1 mmol/L) and/or taking medications for diabetes. BMI: body mass index; CI: confidence interval.

## Data Availability

The datasets supporting the conclusions of this article are available in the University Hospital Medical Information Network Individual Case Data Repository. Please contact the corresponding author to access the data.
